# Nanotechnology for Natural Medicine: Formulation of Neem Oil Loaded Phospholipid Vesicles Modified with Argan Oil as a Strategy to Protect the Skin from Oxidative Stress and Promote Wound Healing

**DOI:** 10.3390/antiox10050670

**Published:** 2021-04-25

**Authors:** Maria Letizia Manca, Maria Manconi, Maria Cristina Meloni, Francesca Marongiu, Mohamad Allaw, Iris Usach, Josè Esteban Peris, Elvira Escribano-Ferrer, Carlo Ignazio Giovanni Tuberoso, Gemma Gutierrez, Maria Matos, Mansureh Ghavam

**Affiliations:** 1 Department of Life and Environmental Sciences, University of Cagliari, Via Ospedale 72, 09124 Cagliari, Italy; mlmanca@unica.it (M.L.M.); mariacristina.meloni@unica.it (M.C.M.); fmarongiu@unica.it (F.M.); allaw.mohamad.22@gmail.com (M.A.); tuberoso@unica.it (C.I.G.T.); 2 Department of Pharmacy and Pharmaceutical Technology and Parasitology, University of Valencia, Burjassot, 46100 Valencia, Spain; iris.usach@uv.es (I.U.); Jose.E.Peris@uv.es (J.E.P.); 3 Biopharmaceutics and Pharmacokinetics Unit, Institute for Nanoscience and Nanotechnology, University of Barcelona, 08007 Barcelona, Spain; eescribano@ub.edu; 4 Department of Chemical and Environmental Engineering, University of Oviedo, 33003 Oviedo, Spain; gutierrezgemma@uniovi.es (G.G.); matosmaria@uniovi.es (M.M.); 5 Department of Range and Watershed Management, Faculty of Natural Resources and Earth Sciences, University of Kashan, Kashan 8731753153, Iran; mansurehghavam@gmail.com

**Keywords:** liposomes, hyalurosomes, keratinocytes, fibroblasts, skin diseases, viscosity, oxidative stress

## Abstract

Neem oil, a plant-derived product rich in bioactives, has been incorporated in liposomes and hyalurosomes modified by adding argan oil and so called argan-liposomes and argan-hyalurosomes. Argan oil has also been added to the vesicles because of its regenerative and protective effects on skin. In the light of this, vesicles were specifically tailored to protect the skin from oxidative stress and treat lesions. Argan-liposomes were the smallest vesicles (~113 nm); the addition of sodium hyaluronate led to an increase in vesicle size (~143 nm) but it significantly improved vesicle stability during storage. In vitro studies confirmed the free radical scavenging activity of formulations, irrespective of their composition. Moreover, rheological investigation confirmed the higher viscosity of argan-hyalurosomes, which avoid formulation leakage after application. In vitro studies performed by using the most representative cells of the skin (i.e., keratinocytes and fibroblasts) underlined the ability of vesicles, especially argan-liposomes and argan-hyalurosomes, to counteract oxidative stress induced in these cells by using hydrogen peroxide and to improve the proliferation and migration of cells ensuring the more rapid and even complete closure of the wound (scratch assay).

## 1. Introduction

In the last decades, the use of natural products for the treatment of human disorders has gained new interest in the scientific community, especially after the discovery of novel natural drugs and their utilization as new therapies for the treatment of human diseases [1]. This stimulated the researchers to continue the search for natural drugs valorising traditional knowledge on herbal medicine [2].

Neem oil has been used since ancient times in the popular medicine thanks to its wide spectrum of biological activities such as antioxidant, antinflammatory, antibacterial [3], antifungal and antiparasitic [4,5]. It is obtained from the neem tree belonging to the family of the Meliaceae (*Azadirachta indica* A.Juss.) and appears as a low-viscous greenish liquid. It is characterized by a pungent smell and is rich in secondary metabolites such as glycerides, fatty acids, sulphur-containing compounds and flavonoids (e.g., quercetin, kaempferol and muricetine), which can exert the above mentioned beneficial effects [6]. This oil may neutralize free radicals and reactive oxygen species and reduce the expression of pro-inflammatory agents involved in the inflammatory process [7,8,9]. Some studies reported antitumor and chemo-preventive effects of neem oil in different types of cancer thanks to its ability to balance redox reactions and to stimulate the host immune responses [10]. Further, in traditional Indian medicine it is used for its moisturizing, antiaging and regenerative properties, mainly connected with its lipophilic composition, fatty acid content (e.g., oleic, palmitic and stearic acids) and antioxidant potential [4]. It is considered a convenient and effective plant-derived oil for wound healing in animals [11]. Indeed, in a previous study, topical use of neem oil associate to the oral administration of curcuma has shown to be effective in the treatment of chronic non-healing wounds [12]. Skin wounds, both acute and chronic, especially those associated with other pathologies like diabetes, leprosy, and peripheral vascular diseases, are still a serious problem for patients, families and clinicians [13]. Many efforts have been made to find effective treatments and in the last decades, several alternative formulations have been designed, including combining nanotechnological systems and natural bioactives [14]. Nanocarriers appear to be a suitable strategy for promoting the delivery of neem oil in the skin, thus improving its antioxidant and wound healing effect. Among the different nanocarriers, phospholipid vesicles are highly versatile and particularly suitable for local application on the skin [15]. To this purpose, they have been specifically modified with different kinds of molecules, such as water co-solvents, surfactants and polymers [16,17,18,19,20,21,22,23,24,25]. These additives improved the carrier performances of vesicles and their skin delivery ability [26]. In particular, sodium hyaluronate and the resulting hyalurosomes were capable of potentiating the wound healing effect of bioactives such as curcumin and liquorice extract [24,27]. Vesicles have been also modified by using different oils as additives capable of modifying the assembling of lipid bilayer. As an example, *Santolina insularis* (Gennari ex Fiori) Arrigoni essential oil, added to hydrogenated phosphatidylcholine generated special vesicles, named santosomes, have promising performances for the treatment of skin lesions [28]. Argan oil enriched liposomes have a softening and relaxing effect on the skin, thus facilitating the payload accumulation and its passage into and through it [29]. Moreover, essential oils such as *Thymus capitatus* essential oil [30], clove essential oil [31], *Zanthoxylum tingoassuiba* essential oil [32], pompia essential oil [33] and others [34], have been loaded in phospholipid vesicles, aiming at improving the oil biological efficacy.

In this study neem oil has been loaded in argan-liposomes and argan-hyalurosomes. Argan oil and sodium hyaluronate were selected as additives, due to their well-known skin care properties [29,35,36,37]. Liposomes without argan oil were prepared as well and used as a reference.

The size, zeta potential and entrapment efficiency of vesicles were measured. The stability of vesicles with regard to pH value, evaporation rate and viscosity changes have been evaluated over time, at different temperatures (4, 25 and 40 °C). The antioxidant activity of neem oil in dispersion or loaded in vesicles was measured in vitro by means of both total reducing power and free radical scavenging. The biocompatibility and protective effect of the formulations against oxidative stress induced in keratinocytes and fibroblasts, along with their ability to stimulate proliferation and cell migration (scratch test), has been tested.

## 2. Materials and Methods

### 2.1. Materials

Soy lecithin and neem oil were purchased from Galeno srl (Carmignano, Prato, Italy). Sodium hyaluronate was purchased by DSM Nutritional Products AG Branch Pentapharm (Dornacherstrasse 112 CH-4147 Aesch BL/Switzerland). Argan oil, 3-(4,5-dimethylthiazol-2-yl)-2,5-diphenyltetrazolium bromide (MTT), gallic acid, ferrous sulphate, 1,1-diphenyl-2-picrylhydrazyl radical (DPPH), (±)-6-hydroxy-2,5,7,8-tetramethylchroman-2-carboxylic acid (Trolox), 2,4,6-tris(2-pyridyl)-1,3,5-triazine (TPTZ), 2,2′-azino-bis(3-ethylbenzothiazoline-6-sulphonic acid (ABTS), neocuproine (2,9-dimethyl-1,10-phenanthroline) hydrochloride, Folin-Ciocalteu’s reagent, sodium carbonate, ferric chloride, ammonium acetate, copper chloride dihydrate, potassium persulphate, copper sulphate pentahydrate and all the other reagents were of analytical grade and were purchased from Sigma-Aldrich (Milan, Italy). Ultrapure water (18 MΩ·cm) was obtained with a Milli-Q Advantage A10 System apparatus (Millipore, Milan, Italy). Reagents and plastics for cell culture were purchased from Life Technologies Europe (Monza, Italy).

### 2.2. Preparation of Phospholipid Vesicles

Liposomes, argan-liposomes and argan-hyalurosomes have been prepared by direct sonication, avoiding the use of organic solvents [38]. For this purpose, soy lecithin (60 mg/mL) and neem oil (2.5, 5 and 10 mg/mL) were placed in glass vials and hydrated overnight with water, to obtain liposomes. Argan oil (5 mg/mL), neem oil (2.5, 5 and 10 mg/mL) and phospholipid were mixed together and hydrated with water to obtain argan-liposomes or were hydrated with a dispersion of sodium hyaluronate (10 mg/mL) in water to obtain argan-hyalurosomes [27,29,37]. The resulting dispersions were sonicated (25 cycles, 5 s ON and 2 s OFF), using a Soniprep150 sonicator (MSE Crowley, London, UK) to obtain small and homogeneously dispersed vesicles [39].

### 2.3. Characterization of Phospholipid Vesicles

Formation and morphology of vesicles were evaluated by cryo-TEM observation. A thin film of each sample was formed on a holey carbon grid and vitrified by plunging (kept at 100% humidity and room temperature) into ethane maintained at its melting point, using a Vitrobot (FEI Company, Eindhoven, The Netherlands). The vitreous films were transferred to a Tecnai F20 TEM (FEI Company), and the samples were observed in a low-dose mode. Images were acquired at 200 kV at a temperature of ~−173 °C, using a CCD Eagle camera (FEI Company) [40].

Average hydrodynamic diameter and polydispersity index of each sample was evaluated by Photon Correlation Spectroscopy by using a Zetasizer nano (Malvern Instruments, Worcestershire, UK). The zeta potential was measured by means of M3-PALS method (phase analysis light scattering) using a Zetasizer nano. All the measurements were performed after dilution of the samples with water [41].

### 2.4. Purification of Vesicles and Evaluation of the Entrapment Efficiency

To evaluate the amount of phytochemicals loaded into the vesicles, samples (2 mL) were purified by dialysis (Spectra/Por^®^ 172 membranes: 12–14 kDa 173 MW cut-off, 3 nm pore size; Spectrum Laboratories Inc., DG Breda, The Netherlands) against water (2 L) for 2 h at room temperature (~25 °C), refreshing the water after 1 h to allow the complete removal of the non-entrapped bioactives. At the end of the purification process, the antioxidant activity of the samples, before and after dialysis, was measured by means of the DPPH assay, and the entrapment efficiency was calculated as a percentage of the antioxidant activity measured after dialysis versus that detected before dialysis. Briefly, neem oil dispersion or vesicle dispersions have been diluted (1:50) with a methanolic solution of DPPH (4 μg/mL). The same dilution (1:50) was made for the DPPH solution used as a control. After dilutions, the samples were kept at room temperature for 30 min in the dark and were subsequently analysed at a wavelength of 517 nm by using a microplate reader (Synergy 4, Synergy ™ Multi-Detection Microplate Reader, Bio-Tek Instruments, AHSI SPA, Bernareggio, Italy). The antioxidant power before and after dialysis of the formulations has been calculated according to the formula reported below [39,42]:

Antioxidant activity (AA%) = [(ABS_DPPH_ − ABS_sample_)/ABS_DPPH_] × 100

### 2.5. Stability Studies of Phospholipid Vesicles

A stability study was carried out by monitoring the average hydrodynamic diameter and the polydispersity index of the vesicles stored at room temperature (~25 ± 1 °C) for a period of 90 days.

The stability of the vesicles was also evaluated by means of static multiple light scattering using the Turbiscan Lab Expert (Formulaction, l’Union, France) [43]. Samples (without dilution) were placed in the test cell, and backscattered light was monitored as a function of time and cell height for 28 days at 30 °C using an Ageing Station (Formulaction). The optical reading head scanned the sample in the cell, providing Transmission (TS) and Backscattering (BS) data every 40 μm in % relative to standards (suspension of monodisperse spheres and silicone oil), as a function of sample height (in mm). The obtained profiles build up a macroscopic fingerprint of the sample at a given time, providing useful information about changes in droplet size distribution, or appearance of a creaming layer, or a clarification front with time. For a comparative evaluation between the different samples we evaluated the Turbiscan Stability Index (TSI) computation as well, that provides a key number related to the general behaviour of the formulation.

### 2.6. Determination of Total Reducing Power (FRAP and CUPRAC Assays), Free Radical Scavenging Activity (DPPH^•^ and ABTS^•+^ Assays) and Folin-Ciocalteu’s Assay

All the assays were performed spectrophotometrically by diluting the samples with methanol (1:40, *v*/*v*) and using a Cary 50 spectrophotometer (Varian, Leinì, TO, Italy). The FRAP assay was assessed preparing a ferric complex of 2,4,6-tris(pyridin-2-yl)-1,3,5-triazine (TPTZ) and Fe^3+^ (0.3123 g TPTZ, 0.5406 g FeCl_3_·6H_2_O in 100 mL acetate buffer pH 3.6). Next, 20 μL of the extract was dissolved in 2 mL of ferric complex and, after an incubation period of 4 min in the dark, absorbance at 593 nm was measured [44]. CUPRAC assay was performed according to the procedure developed by Bektaşǒglu et al. [45]. Briefly, 100 μL of sample were dissolved in a mixture of 500 μL of 10 mM CuCl_2_ solution in water, 500 μL of 7.5 mM neocuproine solution in methanol and 500 μL of 1.0 M CH_3_COONH_4_ buffer at pH  =  7.0. After an incubation period of 30 min in the dark, absorbance at 450 nm was measured. For both FRAP and CUPRAC assays results were expressed as mmol/L of Fe^2+^ obtained from a FeSO_4_ solution having an antioxidant capacity equivalent to that of the dilution of the formulations. The procedure for the development of the DPPH^•^ assay was previously detailed by Tuberoso et al. [44]. This assay was performed to measure the ability of the antioxidants to scavenge the radical cation 1,1-diphenyl-2-picrylhydrazyl radical. 50 μL of the extract was dissolved in 2 mL of 0.06 mmol/L DPPH^•^ in methanol. Then, spectrophotometric readings were carried out at 517 nm after an incubation period of 60 min at room temperature in the dark. A calibration curve in the range of 0.02–1.0 mmol/L was prepared for Trolox, and the data were expressed as Trolox equivalent antioxidant capacity (TEAC mmol/L). The ABTS^•+^ assay was performed according to Re et al. [46], with some modifications [44]. The ABTS^•+^ cation radical was produced by the reaction between 10 mL of 2 mM ABTS in water and 100 μL of 70 mM potassium persulfate, stored in the dark at room temperature for 24 h. The ABTS^•+^ solution was then diluted with methanol to obtain an absorbance of 0.70  ±  0.02 at λ  =  734 nm, and was equilibrated at 30 °C. Samples were prepared in triplicate by diluting 20 μL of samples in 2 mL of the ABTS^•+^ solution diluted with methanol. After 1 min of reaction, absorbances were recorded at 734 nm. DPPH^•^ and ABTS^•+^ data were reported as Trolox equivalent antioxidant capacity (TEAC, mmol/L) obtained from a Trolox solution having an antiradical capacity equivalent to that of the dilution of the formulations. The Folin-Ciocalteu’s assay was performed according to the procedure developed by Tuberoso et al. [44] and the results were expressed as mg/L of gallic acid equivalent (GAE). Briefly, 100 µL of diluted sample was mixed with 500 µL Folin-Ciocalteu reagent, then after 5 min, 3 mL of 10% Na_2_CO_3_ (*w*/*v*) were added. The mixture was stirred, diluted with water to a final volume of 10 mL, and then left for 90 min incubation period at room temperature. The absorbance was read at 725 nm against a blank.

### 2.7. Measurements of pH during the Storage

Measurements of pH were performed using a pH meter Mettler Toledo (Mettler Toledo S.p.A., Milan, Italy). The pH-meter was calibrated using two standard buffer solutions (pH 7 and 4). All measurements were performed at room temperature (25 °C). pH was monitored for one week to evaluate the stability of the samples over time.

### 2.8. Measurements of Water Loss on Storage

The stability of the dispersions was evaluated as a function of water evaporation. Each sample (0.2 g) has been transferred in glass tubes and maintained, for 7 days, at different temperatures: 4, 25 and 40 °C. Every day the samples were weighed to quantify the water loss.

### 2.9. Measurements of Viscosity during the Storage Period

Viscosity studies have been carried out by using a Brookfield Programmable LVDV-II + Viscometer (AMETEK GB LTD T/A Brookfield Technical Centre, Essex, UK), connected to a FE2 HAAKE thermostated bath. The viscosity of the dispersions at given shear rates and controlled temperature (~25 ± 2 °C) has been measured. Each rotation speed (RPM, revolutions per minute) was held constant for 30 s. Different rotation speeds were used (2-4-6-8-10-20-30-40-60-80-100-120-140-160-180-200 RPM) and the viscosity (mPas) as a function of Torque (torsion force expressed as a percentage), Shear Rate (1/s) and Shear Stress (dyne/cm^2^) were measured.

### 2.10. Cytotoxicity of Vesicles against Keratinocytes and Fibroblasts

Keratinocytes (HaCat) and fibroblasts (3T3) (ATCC collection, Manassas, VA, USA), were grown as monolayers in 75 cm^2^ flasks, incubated at 37 °C with 100% humidity and 5% CO_2_. Dulbecco’s Modified Eagle Medium (DMEM) with high glucose, supplemented with 10% foetal bovine serum, 1% penicillin and streptomycin, and 0.1% fungizone, was used to culture the cells. The medium was changed every two days to ensure cell growth.

Cytotoxicity of neem oil in dispersion or loaded in vesicles has been evaluated by the colorimetric MTT (tetrazolium salt, 3- (4,5-dimethylthiazol-2-yl) -2,5-diphenyltetrazolium bromide) test, which is based on the ability of the MTT compound to be metabolized by a mitochondrial enzyme, succinate dehydrogenase. The reduction of MTT leads to the formation of blue-violet crystals of formazan, insoluble in water. Alive cells are able to reduce MTT and the amount of formazan produced is proportional to the number of viable cells [29].

Briefly, keratinocytes and fibroblasts (7500 cells/well) were seeded in 96-well plates, cultured for 24 h, and then exposed for 48 h to the samples properly diluted with medium to achieve the desired concentration of neem oil (20, 10, and 1 μg/mL). Thereafter, MTT solution (0.5 mg/mL final concentration) was added to each well, and 3 h later was removed, replaced with dimethyl sulfoxide, and the absorbance of the solubilized dye was read at 570 nm with a microplate reader (Multiskan EX, Thermo Fisher Scientific Inc., Waltham, MA, USA). The results are shown as a percentage of live cells in comparison with untreated control cells (100% cell viability). Experiments were performed in triplicate (n = 8).

### 2.11. Protective Effect of Vesicles against Stress Induced with Hydrogen Peroxide in Cells

To evaluate the effectiveness of the formulations against oxidative damages induced with hydrogen peroxide, the cells were seeded into 96-well plates, incubated for 24 h, stressed with hydrogen peroxide (1:50,000 dilution) and simultaneously treated with neem oil in dispersion or loaded in vesicles (10 µg/mL). After 4 h, the cells were washed with PBS, and the cell viability was measured using the MTT assay. Untreated cells (100% viability) were used as a positive control, and cells stressed with hydrogen peroxide and untreated with formulations, were used as a negative control. Experiments were performed in triplicate (n = 8).

### 2.12. Scratch Assay

The ability of the neem oil in dispersion or loaded in vesicles to promote proliferation and migration of keratinocytes and fibroblasts and the wound re-epithelization was evaluated using a scratch assay model. Cells were cultured in 6 well plates until a complete monolayer was reached. Then a linear wound was generated using a sterile plastic pipette tip. The scattered fragments of cells were removed by gently washing with fresh medium (DMEM). Cells were then treated with formulations (10 μg/mL of neem oil) and incubated for 24 and 48 h. Untreated cells were used as negative control. At each time point cell monolayers were observed using an optical microscope with a 10x objective. Images at time zero (t = 0 h) were captured to record the initial area of the wounds, and the recovery of the wounded monolayers was measured at 12, 24, 36, and 48 h (t = Δ h). The captured images were quantified by Java’s image J software (1.8.0_172, http://rsb.info.nih.gov, accessed on 1 September 2020) by measuring the area of the wound [47]. The migration of cells toward the wounds was expressed as percentage of wound closure:

wound closure % = [(a_0_ − a_∆_/a_0_] × 100

where a_0_ is the wounded area immediately after scratching, and a_∆_ is the wounded area measured at 12, 24, 36, and 48 h after scratching.

### 2.13. Statistical Analysis of Data

The results were expressed as mean value ± standard deviation. Statistically significant differences among samples were determined by using variance analysis. The post hoc Tukey–Kramer *t*-test was used to substantiate a significant difference between the means of two specific groups. The statistical analysis was performed by using the Excel software package (Microsoft Corp, Redmond, WA, USA) equipped with a tool for statistical analysis. The minimum level of significance chosen was *p* < 0.05.

## 3. Results

### 3.1. Preparation and Characterization of Vesicles

In order to mask the disagreeable smell and flavour and to improve skin bioavailability, neem oil was incorporated in liposomes, argan-liposomes and argan-hyalurosomes. A pre-formulation study was performed using an increasing amount of oil (2.5, 5, 10, 20 mg/mL) aiming at selecting the highest amount that could be incorporated. Using 20 mg/mL of oil, dispersions were unstable, and a separation phase was observed immediately after sonication. Then, only the lower three concentrations (2.5, 5, 10 mg/mL) were used, and the physicochemical characteristics of the prepared vesicles were measured (Table 1). Liposomes were ~140 nm in size, polydispersed (~0.35), and highly negatively charged (~−65 mV) irrespective of the concentration of oil loaded into them. The addition of argan oil led a decrease of both mean dimeter and polydispersity index, while the zeta potential remained unchanged. The addition of argan oil and hyaluronan did not significantly affect the vesicle size and zeta potential as these values were like that of liposomes. Only a slight decrease of polydispersity index was observed.

All vesicles had a strongly negative surface charge, regardless of their composition, which is predictive of a good stability of the system over time (Table 1).

Liposomes, argan-liposomes and argan-hyalurosomes were able to incorporate high amounts of neem oil, as the entrapment efficiency was always higher than 60%, without significant differences between samples. However, the effective concentration of neem oil entrapped inside the vesicles increased as the amount of neem oil increased as in 10liposomes, 10argan-liposomes and 10argan-hyalurosomes it was ~6.6 mg/mL.

**Figure 1:**
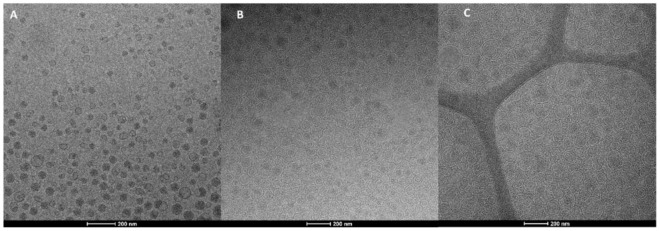
Representative cryo-TEM images of 10liposomes (**A**), 10argan-liposomes (**B**) and 10argan-hyalurosomes (**C**).

Cryo-TEM images confirmed the formation of vesicles. The morphology of vesicles was not affected by the used neem oil concentration but mostly by the presence of vesicle additives (argan oil and hyaluronan). Indeed, liposomes are mainly unilamellar while the lamellarity increased upon the addition of argan oil or the combination of argan oil and sodium hyaluronate. Argan-liposomes and argan-hyalurosomes were mainly oligolamellar and close-packed vesicles (Figure 1) [48].

### 3.2. Stability Studies

**Figure 2:**
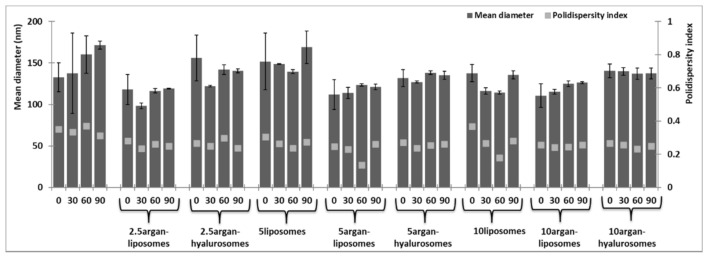
Average hydrodynamic diameter and polydispersity index of liposomes, argan-liposomes and argan-hyalurosomes loading increasing amount of neem oil over 90 days of storage. Mean values ± standard deviations (error bars) have been reported.

The monitoring of the average hydrodynamic size and the polydispersity index of vesicles for 90 days at 25 °C, disclosed that 2.5liposomes, 2.5argan-hyalurosomes and 5liposomes were not very stable as their size increased during the storage (Figure 2). Other samples, especially those loading 10 mg/mL of neem were stable and the mean hydrodynamic diameter and polydispersity index did not change under storage. As previously reported, both argan oil and sodium hyaluronate were confirmed to be considered as key components in improving the stability of the vesicles against aggregation and fusion phenomena [24,27,29].

The long-term stability of liposomes, argan-liposomes and argan-hyalurosomes has been better evaluated by the TurbiscanTM technology. This method provides, in a short time, information on possible destabilization processes occurring in a colloidal dispersion, such as reversible particle migration (sedimentation, flocculation, or creaming) and irreversible particle-size change (coalescence).

**Figure 3:**
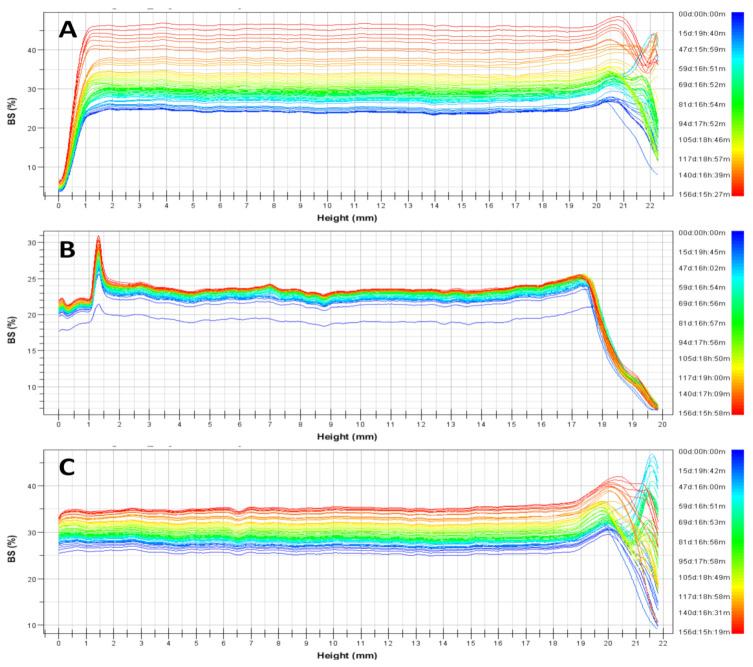
Representative backscattering profiles and Turbiscan Stability Index (TSI) of liposomes (**A**), argan-liposomes (**B**), and argan-hyalurosomes (**C**).

The behaviour of dispersions was similar irrespective to the used concentration of neem oil (Figure 3). Any important modifications of backscattering profiles were observed for argan-liposomes and argan-hyalurosomes, indicating that no coalescence, sedimentation, flocculation or clarification occurred. Higher modifications of backscattering profiles were observed for liposomes, denoting a lower stability of the system over time. Indeed, the Turbiscan Stability Index, which corresponds to a cumulative sum of all the backscattering or transmission variation of the entire sample, was significantly higher in comparison with that of argan-liposomes and argan-hyalurosomes.

### 3.3. pH Measurements

The pH value of vesicles was ~6 irrespective of their composition and it was slightly reduced over time (30 days) but it was never lower than ~5 (data not shown). These results are in agreement with those obtained by monitoring size and polydispersity index, as the maintenance of the pH values is also predictive of good stability, confirming that degradation phenomena (fusion or aggregation) are completely avoided. Indeed, it is well known that pH may modify the structure and shape of vesicles, which may completely dissociate at pH below 4 [49].

### 3.4. Evaluation of the Capability of Vesicles to Avoid Water Loss

**Figure 4:**
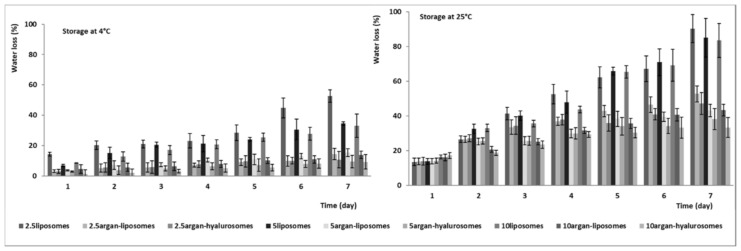
Amount of water loss (%) from liposomes, argan-liposomes and argan-hyalurosomes over 7 days of storage at 4 °C and 25 °C. Mean values ± standard deviations (error bars) have been reported.

The amount of water evaporated from the dispersions has been measured at 4, 25 and 40 °C (Figure 4). Clearly, the lowest values were obtained at 4 °C and the water loss increased as a function of the temperature. Indeed, at 40 °C, the water loss was completed (100%) after the second day of storage (data not shown). The water loss at 4 and 25 °C was affected by vesicle composition. Liposomes lost the higher amount of water, and the loss decreased when the concentration of neem oil increased. It was ~50% for 2.5liposomes and ~35% for 5liposomes and 10liposomes at 7 days and 4 °C; ~90% for 2.5liposomes and ~85% for 5liposomes and 10liposomes at 7 days and 25 °C. The water loss of argan-liposomes and argan-hyalurosomes was significantly reduced by the presence of argan oil. It was ~10% at 4 °C and ~40% at 25 °C irrespective of the concentration of neem oil used. Results underline a strong correlation between higher oil content and higher water retention. This is probably because the high amount of oil allowed the formation of a more impermeable lipophilic bilayer, which better prevents the loss of water encapsulated inside the vesicles.

### 3.5. Rheological Studies

**Figure 5:**
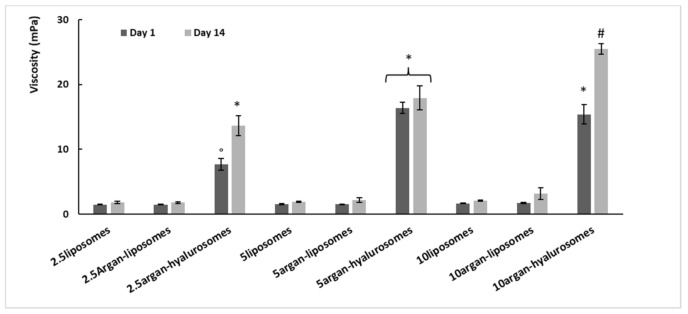
Viscosity values of liposomes, argan-liposomes and argan-hyalurosomes measured at 200 rpm, at day 1 and 14 of storage. Mean values ± standard deviations (error bars) have been reported. Symbols (*, °, ^#^) indicate values statistically different (*p* < 0.01).

The viscosity of the samples has been measured at 200 rpm and 25 °C at day 1 and 14 of storage (Figure 5). Viscosity of liposomes and argan-liposomes was ~2 mPas and was not affected by neem oil concentration (2.5, 5, 10 mg/mL) and storage time (1 and 14 days). The viscosity of argan-hyalurosomes was higher: ~9 mPas that of 2.5argan-hyalurosomes and ~15 mPas that of 5 and 10argan-hyalurosomes. The viscosity of argan-hyalurosomes was higher at 14 days of storage, probably due to the loss of water in the intervesicle medium. The addition of sodium hyaluronate caused a significant increase in viscosity, probably due to the ability of sodium hyaluronate to form a stable network surrounding the vesicles [27].

### 3.6. Antioxidant Activity of Formulations

The antioxidant power and total phenolic content of formulations were measured using different assays (Table 2). FRAP and CUPRAC assays have been used to estimate the total reducing power and DPPH^•^ and ABTS^•+^ assays to assess the free radical scavenging activity. The Folin-Ciocalteu assay is traditionally used to estimate the total phenolic content, but being based on transfer of electrons, it measures the reducing capacity of the samples as well. A statistically significant linear relationship (r ≥ 0.9, *p* ≤ 0.01) was observed among the sets of results for the five assays. It can be noticed that by increasing the amount of neem oil the antioxidant power also increased, although this increase was statistically significant (*p* ≤ 0.05) only for vesicles loading 10 mg/mL of neem oil irrespective of the assay performed. The presence of argan oil or sodium hyaluronate affected the results but a clear antioxidant contribution was not clearly detected.

### 3.7. In Vitro Toxicity Studies on Keratinocytes and Fibroblasts

**Figure 6:**
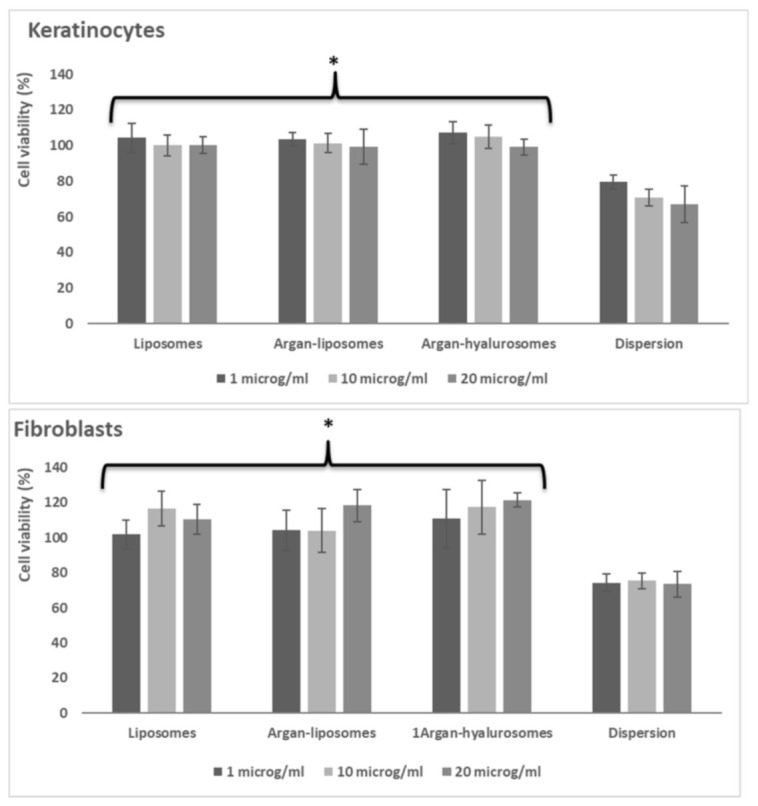
Viability of keratinocytes and fibroblasts incubated for 48 h with neem oil in dispersion or loaded in liposomes, argan-liposomes and argan-hyalurosomes at 3 different dilutions (1, 10 and 20 μg/mL of neem oil). Symbol (*) indicates the same value significantly different from the neem oil dispersion (*p* < 0.01).

Further studies were carried out to evaluate the biological activity of oil load vesicles. To this purpose, considering their higher potential, liposomes, argan-liposomes and argan-hyalurosomes loading 10 mg/mL of neem oil were used. At first screening, the biocompatibility of formulations was evaluated measuring the viability of keratinocytes and fibroblasts after incubation with formulations, properly diluted with cell medium to obtain three different concentrations: 1, 10 and 20 μg/mL of neem oil. Cell viability was measured at 48 h using the MTT cell viability test (Figure 6). Any toxicity was detected after treatment of cells (keratinocytes and fibroblasts) with vesicle dispersions, irrespective of their composition, while the incubation of cells with neem oil in dispersion caused 30% of cell apoptosis, regardless of the concentration used.

Considering the non-toxicity of formulations, the intermediate dilution (10 μg/mL of neem oil) was used to perform further studies.

### 3.8. Protective Effect of Formulations against Oxidative Stress Induced in Keratinocytes and Fibroblasts by Using Hydrogen Peroxide

**Figure 7:**
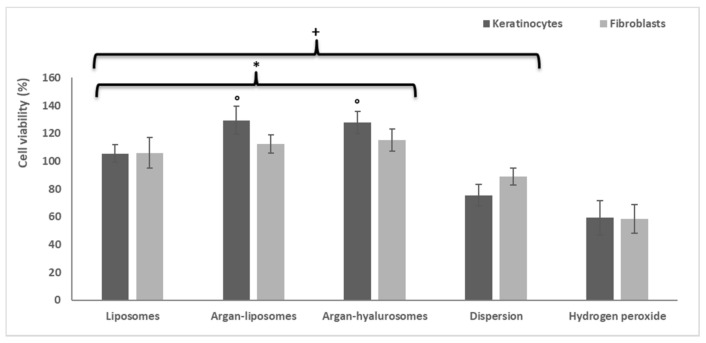
Protective effect of neem oil formulations under oxidative stress induced with hydrogen peroxide in keratinocytes and fibroblasts. Symbol (+) indicates values significantly different from that of cells stressed with hydrogen peroxide (*p* < 0.05); symbol (*) indicates values significantly different from that of cells treated with neem oil dispersion (*p* < 0.05) and symbol (°) indicate values significantly different from that of cells treated with liposomes.

Keratinocytes and fibroblasts were exposed to oxidative stress using hydrogen peroxide and were simultaneously treated with neem oil in dispersion or loaded in vesicles (Figure 7). The viability of cells treated with hydrogen peroxide was ~58% for both keratinocytes and fibroblasts, as it is well known that hydrogen peroxide may cause their apoptosis and death. The treatment with the neem oil dispersion reduced the damaging effect of hydrogen peroxide and the cell viability slightly increased up to ~75% for keratinocytes and ~89% for fibroblasts (*p* < 0.05 versus the viability of stressed and untreated cells). The treatment with liposomes allowed normal conditions to be restored and reach ~100% viability of both keratinocytes and fibroblasts (*p* < 0.05 versus viability of cells treated with dispersion or untreated). The treatment with argan-liposomes and argan-hyalurosomes also inhibited the damaging effect of hydrogen peroxide, permitting fibroblast viability to reach ~100% (*p* < 0.05 versus viability of cells treated with dispersion or untreated) and keratinocyte viability ~120%.

**Figure 8:**
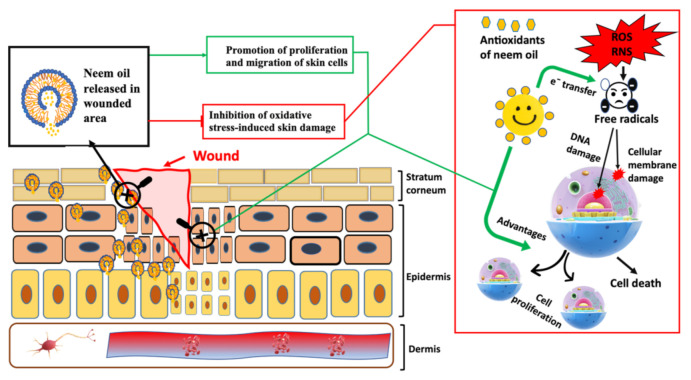
Schematic representation of the antioxidant activity of neem oil containing vesicles.

The results confirmed that the loading of neem oil in vesicles, especially argan-liposomes and argan-hyalurosomes, improved the effectiveness of bioactives to protect keratinocytes and fibroblasts from damage caused by oxidative stress, Figure 8.

### 3.9. Scratch Assay

**Figure 9:**
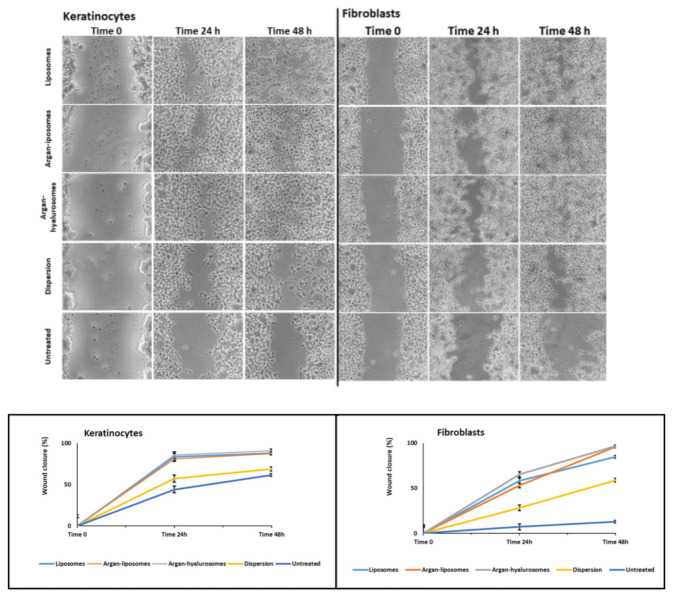
Representative images of scratch and percentage of wound closure in keratinocytes and fibroblasts, untreated or treated with neem oil in dispersion or loaded in liposomes, argan-liposomes and argan-hyalurosomes.

The scratch assay was performed in vitro using a single cell layer of keratinocytes and fibroblasts. The effectiveness of formulations in promoting wound closure was evaluated by measuring the thickness of the scratch over time (Figure 9). At 24 h the wound closure of keratinocytes treated with neem oil dispersion was ~40%, slightly higher than that obtained with untreated cells, and at 48 h did not further improve. The wound closure provided by the treatment with neem oil loaded liposomes, argan-liposomes and argan-hyalurosomes was ~85% at 24 h and reached ~100% at 48 h. The proliferative and migrative effect induced by neem oil loaded vesicles was evident also in the fibroblast assay but to a lesser extent. At 48 h, wound closure of untreated fibroblasts was only ~20% and that of fibroblasts treated with neem oil in dispersion was ~40%. Using neem oil loaded liposomes, argan-liposomes and argan-hyalurosomes was ~55% at 24 h and reached ~95% at 48 h. According to previous findings, neem oil loaded vesicles were more effective in promoting proliferation and migration of keratinocytes than fibroblasts.

## 4. Discussion

Plant-derived bioactives are safe and effective molecules with valuable biological efficacy, often higher than that obtained by using synthetic drugs [50]. Many active molecules have been extracted from different plants, isolated and used as pure bioactive or as a phytocomplex [51]. Indeed, the phytocomplexes seem to be more effective as they contain different active components, which may often exert synergistic activities [52]. However, the potential use of plants as a source of new drugs is still poorly explored and their effective clinical use is limited by the dissipative extraction and purification procedures needed to isolate the compounds of interest, the low yield and the low accessibility of matrices [51,53]. In addition, the information and scientific studies are often incomplete or not-available, mainly because an effective evaluation would be very expensive and depend on the integration of several sciences such as botany, phytochemistry, pharmaceutical technology, pharmacology and toxicology [54].

Many efforts have been made to evaluate the therapeutic activity of these natural molecules [55]. Despite their promising therapeutic properties, these natural drugs are often low water-soluble, poorly bioavailable and metabolically instable, and as a consequence the performed studies did not confirm their effective potential [56]. Therefore, the development of suitable formulations, especially those formulated by using nanotechnological processes, represent a valuable strategy capable of overcoming the various drawbacks, which often occur using natural drugs [57]. This new approach can promote the development of innovative and technological natural-based medications improving the exploitation of traditional medicine. Neem oil has been widely used in traditional medicine for the treatment of various disorders, especially those affecting the skin [58]. However, its use is limited especially because of its low bioavailability along with its characteristic and unpleasant smell, defined as a combination of peanuts and garlic [59]. Phospholipid vesicles have been demonstrated to be very effective in improving the efficacy of drugs in the treatments of skin diseases. Additionally, it has been confirmed that to improve the skin delivery of each molecule or phytocomplex, specific ad hoc formulated vesicles are needed. The use of a co-solvent like glycerol or propylene glycol, among others, may provide a better packing of phospholipid giving the formation of small and deformable systems capable of penetrating and diffusing in a better extent than conventional liposomes into and through the skin [22,60,61]. In previous studies, neem oil has been loaded in liposomes for skin delivery [62]. Alternatively, it was loaded in solid lipid nanoparticles for the treatment of acne or in nanoemulsions [63,64]. In the present study, the neem oil was loaded in liposomes and hyalurosomes, which were in turn modified by adding argan oil [24,65]. Hyalurosomes, has been selected due their performances as topical carriers for natural bioactives [27]. Similarly, the ability of argan oil to promote skin hydration and relaxation, thus promoting the delivery of different payloads has been confirmed in previous studies [29]. To find the highest amount of neem oil that could be loaded inside the vesicles, increasing concentrations were used (2.5, 5, 10, 20 mg/mL). Loading 20 mg/mL the dispersions separated after sonication, then 2.5, 5, 10 mg/mL were used. The entrapment efficiency was always ~68% irrespective of the composition of vesicles, while the highest concentration of loaded neem oil (~6.5 mg/mL) was reached using vesicles prepared with 10 mg/mL of oil. According to previous studies, the addition of an oil may play a key role in the vesicle assembly and properties; indeed argan-liposomes were the smallest while the addition of hyaluronan slightly increased the vesicle size [66]. Argan-liposomes and argan-hyalurosomes were more lamellar and close-packed than liposomes. The addition of argan oil strongly reduced the water loss of dispersions during storage, probably due the formation of a more stable and impermeable lipidic bilayer, which avoids the evaporation of water encapsulated inside the vesicles. The argan oil also contributed to the improvement of vesicle stability, as confirmed by Tusbiscan results, probably thanks to the formation of a well packed bilayer, which traps the vesicles, avoiding aggregation, fusion and precipitation phenomena. Overall, the addition of argan oil to argan-liposomes and argan-hyalurosomes positively affected the vesicle properties, while the concentration of neem oil on these parameters was negligible. In addition, the presence of hyaluronan in argan-hyalurosomes allowed an increase of viscosity, which can facilitate the application of the formulation on the skin, avoiding its leakage and favoring adhesion [67]. Highly viscous systems can be easily spread in the skin and the loss of the formulation is almost completely avoided. After application they can be retained in the site of application and were able to promote the skin hydration causing an alteration of the ordered structure of the stratum corneum and promoting the formation of strategic pathway for the passage of the bioactives alone or still incorporated into the vesicles—especially argan-hyalurosomes [21].

Overall results on physicochemical and technological properties of formulations underlined that the loading of 10 mg/mL of neem oil was the most advantageous concentration for preparing stable and effective phospholipid vesicles. Indeed, using this concentration the total phenolic content and antioxidant power were higher. For this reason, in vitro studies have been performed only using liposomes, argan-liposomes and argan-hyalurosomes loading 10 mg/mL of neem oil.

An important aspect to be considered when studying a new formulations is the evaluation of its safety even before of its effectiveness [68]. For this purpose, in vitro biocompatibility studies have been performed by using the most important cells of the skin: keratinocytes and fibroblasts [69]. Keratinocytes represent the largest epidermal cell population in the skin and regulate the characteristics of the skin surface, essentially through their correct proliferation and differentiation [70]. Fibroblasts are mainly located in the dermis; they produce collagen and elastin to ensure the elasticity and the integrity of the skin [71]. Together these cells are mainly involved in the protection and support of the skin and thus of the entire human body [72]. Biocompatibility studies confirmed the high safety of formulations and underlined the ability of argan-liposomes and argan-hyalurosomes to also promote the proliferation of keratinocytes. Moreover, the damages induced in cells by using hydrogen peroxide were avoided using these formulations, especially argan-liposomes and argan-hyalurosomes. These results might be related to the ability of vesicles to interact with cells promoting the internalization of bioactives [73,74].

Proliferation and migration of keratinocytes and fibroblasts were promoted by treating the wound with neem oil, but the wound closure was completed only by using argan-liposomes and argan-hyalurosomes. The promotion of cell proliferation and migration may be mainly connected with the ability of argan oil to promote the relaxing and softening effect on skin, when used in combination with phospholipid vesicles [29]. Clearly, the performed studies must be confirmed by further and more specific studies, to disclose the role of both argan oil and sodium hyaluronate on the performance of neem oil loaded vesicles.

## 5. Conclusions

The physicochemical and technological studies underlined that 10 mg/mL of neem oil can be used to prepare argan-liposomes and argan-hyalurosomes. The resulting vesicles were sized around 140 nm and negatively charged. The dispersions were stable, and at 25 °C were capable of retaining water during storage. Argan-hyalurosomes were more viscous and seemed to be more suitable for skin application. The biological study confirmed that argan-liposomes and argan-hyalurosomes were highly biocompatible and could effectively protect the skin cells from oxidative stress, improving the efficacy of the oil. In addition, formulations significantly promote the wound closure to a better extent than the oil dispersion. In light of these results, we can conclude that the nanotechnological formulations of neem oil such as argan-liposomes and especially argan-hyalurosomes seem to represent a promising strategy for enhancing the therapeutic effects of the oil following topical administration, giving a natural alternative for the treatment of skin lesions and disorders.

## Figures and Tables

**Table 1:** Mean dimeter (MD), polydispersity index (PI), zeta Potential (ZP) and entrapment efficiency (EE) of liposomes, argan-liposomes and argan-hyalurosomes loading increasing amount of neem oil (2.5, 5, 10 mg/mL). Mean values ± standard deviations have been reported. Each symbol (*, °) indicates the same value *p* > 0.05.

Samples	MD (nm)	PI	ZP (mV)	EE (%)
2.5liposomes	° 133 ± 17	0.35	−75 ± 7	62 ± 13
2.5argan-liposomes	* 118 ± 6	0.28	−77 ± 5	65 ± 3
2.5argan-hyalurosomes	° 156 ± 17	0.26	−75 ± 2	63 ± 4
5liposomes	° 152 ± 14	0.32	−80 ± 6	69 ± 10
5argan-liposomes	* 112 ± 8	0.25	−75 ± 6	71 ± 3
5argan-hyalurosomes	° 133 ± 10	0.27	−73 ± 2	64 ± 4
10liposomes	° 138 ± 10	0.36	−77 ± 4	64 ± 12
10argan-liposomes	* 110 ± 7	0.25	−68 ± 6	66 ± 7
10argan-hyalurosomes	° 140 ± 8	0.26	−73 ± 5	65 ± 7

**Table 2:** Total phenol content and antioxidant power of the formulations. FRAP and CUPRAC values are expressed as Fe^2+^ mmol/l, DPPH^•^ and ABTS^•+^ values are expressed as Trolox equivalent antioxidant capacity (TEAC, mmol/L), total phenol content measured by Folin-Ciocalteu’s assay is expressed as gallic acid equivalent (GAE). Mean values ± standard deviations have been reported. Means in each column not sharing a superscript letter are significantly different (*p* ≤ 0.05).

Samples	FRAP (mmol Fe^2+^/L)	CUPRAC (mmol Fe^2+^/L)	DPPH^●^ (TEAC mmol/L)	ABTS^●+^ (TEAC mmol/L)	TPC (mg GAE/L)
2.5liposomes	2.48 ^a^ ± 0.09	9.71 ^ac^ ± 0.29	0.41 ^a^ ± 0.02	0.46 ^ac^ ± 0.03	0.23 ^ab^ ± 0.03
2.5argan-liposomes	2.26 ^b^ ± 0.11	7.96 ^b^ ± 0.67	0.37 ^a^ ± 0.03	0.37 ^b^ ± 0.01	0.21 ^a^ ± 0.02
2.5argan-hyalurosomes	2.29 ^ab^ ± 0.12	9.26 ^bc^ ± 0.76	0.42 ^ab^ ± 0.03	0.44 ^a^ ± 0.02	0.21 ^a^ ± 0.02
5liposomes	2.37 ^ab^ ± 0.07	9.82 ^c^ ± 0.17	0.49 ^b^ ± 0.04	0.50 ^c^ ± 0.02	0.25 ^ab^ ± 0.03
5argan-liposomes	2.44 ^ab^ ± 0.18	10.92 ^d^ ± 0.73	0.48 ^b^ ± 0.04	0.50 ^c^ ± 0.01	0.25 ^ab^ ± 0.04
5argan-hyalurosomes	3.04 ^c^ ± 0.23	11.81 ^d^ ± 0.46	0.48 ^b^ ± 0.03	0.48 ^ac^ ± 0.03	0.28 ^b^ ± 0.03
10liposomes	3.62 ^d^ ± 0.13	16.15 ^e^ ± 0.35	0.75 ^c^ ± 0.02	0.63 ^d^ ± 0.04	0.36 ^c^ ± 0.01
10argan-liposomes	3.78 ^d^ ± 0.20	16.31 ^e^ ± 0.98	0.68 ^c^ ± 0.05	0.62 ^d^ ± 0.01	0.34 ^c^ ± 0.02
10argan-hyalurosomes	3.91 ^d^ ± 0.26	17.07 ^e^ ± 0.83	0.74 ^c^ ± 0.01	0.75 ^e^ ± 0.04	0.37 ^c^ ± 0.01

## Data Availability

Data is contained within the article.
